# To the Editors of the Medical and Physical Journal

**Published:** 1803-07-01

**Authors:** J. Gaillard

**Affiliations:** No. 11, Vine Street, Piccadilly


### *To the Editors of the Medical and Phyſical Journal,*

GENTLEMEN,

FROM the earliest period of Vaccine Inoculation, I have paid every attention to it so important a discovery appeared to merit; and experienced at the first commencement (as other practitioners with myself must have done) the difficulty of preserving the virus: of course, every method proposed for this purpose was attended to by me, and now I have no hesitation in giving preference to the mode pointed out by Mr. Dennett in your Journal, which I have adopted for twenty months past. No other plan that I have seen or read of, can be put in competition with this, in my opinion, except that lately proposed by Mr. Ring, to which some objections may be made, as being more complicated. Mr. Ring advises a tooth-pick or ivory lancet, instead of a round pointed quill, as described by Mr. Dennett, and inserts it by puncture rather than by slight incision. But every one must be apprized of the difficulty of keeping the point of the tooth-pick long enough in the puncture for the virus to dissolve, if *quite dry*, when a restless child is to be inoculated, which appears to me the reason the lancet so often fails; but from the pliableness of the quill in rubbing it over the incision, this objection is done away. I must beg also to observe, what appears a material objection to the observation of Mr. Ring with regard to his new instrument called the Vaccinator, with which he says, that if well charged, more than *one* person may be inoculated. I believe it would be impossible to adopt this with satisfaction, as, from the first inoculation, some blood must of necessity be left upon the point of the instrument, and few *parents*, under such circumstances, would like to have it employed. But which ever mode gentlemen may prefer, is of little consequence when compared to the certainty of keeping it active by this means, as I understand Mr. Dennett used in February last a single quill charged in June, 1802, which answered perfectly. This must prove of great value to the institutions about to be established, as large quantities may be kept at little trouble and expence.

I have to apologize for troubling you with these remarks, but they have arisen from a conviction of the benefit Medical Gentlemen will derive by attending to the method proposed. I am, &c.

*No.* 11, *Vine Street, Piccadilly,* J. GAILLARD.

*May* 12, 1803.

